# Plinabulin ameliorates neutropenia induced by multiple chemotherapies through a mechanism distinct from G-CSF therapies

**DOI:** 10.1007/s00280-019-03998-w

**Published:** 2019-12-06

**Authors:** James R. Tonra, G. Kenneth Lloyd, Ramon Mohanlal, Lan Huang

**Affiliations:** BeyondSpring Pharmaceuticals, 28 Liberty Street, 39th Floor, New York, NY USA

**Keywords:** Chemotherapy, G-CSF, Neutropenia, Plinabulin, LSK

## Abstract

**Purpose:**

Chemotherapy-induced neutropenia (CIN) increases the risk of infections and mortality in cancer patients. G-CSF therapies are approved for the treatment of CIN, but non-G-CSF therapies are needed to increase efficacy and minimize side effects. Plinabulin is an inhibitor of tubulin polymerization that ameliorates CIN caused in patients by the microtubule stabilizer docetaxel. The present study evaluates the potential of plinabulin to reduce neutropenia induced by chemotherapies of different classes in a manner not dependent on increasing G-CSF.

**Methods:**

The anti-CIN benefits of plinabulin were tested in rodents co-treated with docetaxel, cyclophosphamide or doxorubicin. Effects on G-CSF levels were evaluated in tissues by immunoassay. Flow cytometry was utilized to test treatment effects on femur bone marrow cell counts from immunocompetent mice-bearing orthotopic 4T1 breast cancer tumors.

**Results:**

Plinabulin alleviated neutropenia induced by microtubule stabilizing, DNA cross-linking and DNA intercalating chemotherapies, yet did not affect bone marrow or blood G-CSF levels. The number of lineage^−^/Sca1^+^/c-Kit^+^ (LSK) hematopoietic stem/progenitor cells (HSPC) in murine bone marrow collected 2 days after treatment was not affected by docetaxel monotherapy despite increased plasma G-CSF in this group. LSK cell number was, however, increased when plinabulin was combined with docetaxel, without affecting G-CSF.

**Conclusions:**

Results support the clinical testing of plinabulin as a non-G-CSF-based treatment for CIN associated with chemotherapies of different mechanisms. Results also support HSPC as a focal point for future mechanism-of-action work aimed at understanding the ability of plinabulin to reduce this serious side effect of cytotoxic therapy in cancer patients.

**Electronic supplementary material:**

The online version of this article (10.1007/s00280-019-03998-w) contains supplementary material, which is available to authorized users.

## Introduction

Myelosuppression is the primary toxicity of many chemotherapy regimens, and neutropenia in particular is a frequent and potentially life-threatening complication [1]. Chemotherapies deplete proliferating cells in the bone marrow [2], leading to a reduction in blood absolute neutrophil count (ANC) that can occur in a matter of days due to the short half-life of circulating mature neutrophils (~ 11–12 h in mice and humans) [3, 4]. Patients that develop severe neutropenia are more susceptible to hospitalization and potentially fatal infections, with a risk for febrile neutropenia that increases with neutropenia severity and duration [1, 5]. Furthermore, severe neutropenia often necessitates omitting scheduled chemotherapy administrations, potentially compromising the benefit to cancer patients [5].

The current treatment for myelosuppressive chemotherapy calls for the prophylactic administration of granulocyte colony-stimulating factor (G-CSF)-based therapies such as a longer acting pegylated form of G-CSF, Neulasta^®^ (pegfilgrastim) [5]. G-CSF promotes the survival and proliferation of neutrophil precursors, supports their differentiation into mature neutrophils, and promotes neutrophil egress from the bone marrow [4, 6]. G-CSF therapies significantly reduce the incidence and duration of severe neutropenia in patients, and increase chemotherapy regimen compliance. The prophylactic use of G-CSF agents has limitations in terms of cost, convenience of use, and adverse effects. Although an on-body injector (Onpro^®^) is available for placement on the skin for next day delivery of pegfilgrastim, many patients are still inconvenienced by the need to return to the treatment site 1–3 days after chemotherapy is dosed. G-CSF adverse effects include bone pain, splenic rupture, acute respiratory distress syndrome, allergic reactions including anaphylaxis and allergies to acrylics, sickle cell disorder, glomerulonephritis, capillary leak syndrome, leukocytosis and the potential for tumor growth stimulatory effects on malignant cells [5, 7]. Finally, with more aggressive chemotherapy regimens such as Taxotere^®^ + Adriamycin^®^ + cyclophosphamide (TAC), grade 3 or 4 neutropenia still occurs in > 90% of patients, despite G-CSF prophylactic therapy [8]. To address these issues, novel non-GSF-based treatments for CIN are being sought as alternatives or supplements to G-CSF therapies [7].

Plinabulin (BPI-2358) is a small molecule agent in Phase 3 testing to increase cancer patient survival following positive effects observed in non-small cell lung cancer patients [9] [Mohanlal et al., ASCO-SITC 2017, Abstract 139]. Plinabulin reversibly binds to β-tubulin within the colchicine pocket [10], preventing polymerization into microtubules [11]. Following microtubule disruption, plinabulin exerts diverse cellular effects ranging from direct killing of cancer cells and proliferating endothelial cells [11, 12], to increasing dendritic cell maturation [13]. Importantly, plinabulin significantly reduced CIN in cancer patients when administered within 1 h following treatment with another tubulin-targeted therapy, docetaxel (Taxotere^®^) [9] [Mohanlal et al., ASCO-SITC 2018, Abstract 126]. This effect stands in contrast to the worsening of CIN by combretastatin A4 [14], a small molecule that also binds to the colchicine pocket, but at a site and with kinetics that differ from that of plinabulin [15]. Studies reported here aimed to determine whether plinabulin could act as a broad acting anti-CIN agent with multiple chemotherapies of diverse classes, utilizing a mechanism distinct from approved therapies that increase circulating G-CSF.

## Materials and methods

### Drugs

Plinabulin monohydrate (BeyondSpring Pharmaceuticals) and pegfilgrastim (McKesson) were formulated and diluted, respectively, in 7.1% Tween 80 (Sigma-Aldrich)/25.5% propylene glycol (Fisher Scientific)/67.4% D5 W (5% dextrose in water; Baxter) for dosing. Docetaxel (Accord Health or Winthrop) was formulated in 0.9% saline or 7.5% ethanol/7.5% Tween 80 in D5 W. Cyclophosphamide (Sigma-Aldrich) was formulated in sterile water for injection and doxorubicin (McKesson) was formulated in 0.9% saline (Baxter). The plinabulin dose level (7.5 mg/kg) was selected to be that previously demonstrated to have in vivo efficacy in cancer models [12] and the lowest dose demonstrating significant efficacy against doxorubicin-induced neutropenia evaluated 2 days after dosing (Online Resource 1). Due to the potential for decreased sensitivity of rodent bone marrow to the cytotoxic effects of chemotherapy [16], we did not select chemotherapy dose levels to be equivalent to the dose causing neutropenia in humans on a mg/kg or mg/m^2^ basis. Chemotherapy doses utilized in rodents were screened or selected from the published literature, and these doses were confirmed to cause neutropenia prior to testing the efficacy of plinabulin (data not shown).

### Chemotherapy-induced neutropenia in normal healthy rats

All procedures performed in studies involving animals were in accordance with the ethical standards of the institution at which the studies were conducted. Adult male Crl-CD Sprague–Dawley rats were obtained from Charles River Canada and studies were performed at Charles River Laboratories in Montreal, Canada. Rats were dosed with docetaxel, cyclophosphamide, doxorubicin or appropriate vehicles, intraperitoneally (IP) or by tail vein intravenous (IV) bolus injection. Plinabulin or plinabulin vehicle was administered IP 30–60 min after chemotherapy. Ethylenediaminetetraacetic acid (EDTA)-treated whole blood was collected by jugular venipuncture during dosing, and from the abdominal aorta under anesthesia at the final time point. ANC was measured using an Advia Hematology System.

### Bone marrow hematopoietic cell evaluation in tumor-bearing mice

One million 4T1 murine breast cancer cells (ATCC) in phosphate-buffered saline (PBS) were implanted in the mammary fat pad of female BALB/c mice (Charles River; 9 weeks of age) at Charles River Laboratories (Morrisville, NC, USA). When mean tumor volume reached approximately 290 mm^3^, tumor-bearing mice were randomized into treatment groups by tumor volume and treated with docetaxel or docetaxel vehicle (25 ml/kg total volume) by 15 min IV infusion, followed by a 100 μl PBS flush. Fifteen minutes later, plinabulin or plinabulin vehicle was administered IP twice, 3 h apart. Two days after dosing, red blood cells in bone marrow collected from both femurs were lysed using ammonium–chloride–potassium (ACK) buffer (Life Technologies). Samples were centrifuged and washed twice with PBS. Pellets were suspended in PBS, pH 7.4, at 2 × 10^7^ cells/mL and kept on ice for flow cytometry. Briefly, 100 µL of single cell suspensions was pelleted, resuspended in Live/Dead Aqua (Life Technologies) and stained for 30 min at 4 °C. Cells were then probed with antibody panels for 30 min at 4 °C. Data were collected on a FACSCanto II™ (BD Biosciences) and analyzed with FlowJo software (Tree Star). Primary antibody targets (fluorochrome) included: CD3 (PerCP-Cy5.5), CD45 (APC-Fire750), CD49b (PerCP-Cy5.5), F4/80 (PerCP-Cy5.5) and Ly6G (BV785) (BioLegend), as well as CD11b (BUV395), CD16/32 (BV605), CD19 (PerCP-Cy5.5), CD34 (FITC), CD48 (BUV737), CD115 (PE), CD150 (BV421), c-kit (APC), Flt3 (PE-CF594), IL-7Rα (BV711) and Sca-1 (PE/Cy7) (BD Biosciences). Lineage (Lin) status was evaluated with a combination of probes for CD3, CD19, CD49b and F4/80.

### G-CSF protein measurement

G-CSF was measured by ELISA [R&D Systems] in rat bone marrow samples flushed from the femur with PBS, and total protein was evaluated by bicinchoninic acid assay. G-CSF was measured in EDTA plasma isolated from blood collected by cardiac puncture in anesthetized non-tumor-bearing and 4T1 tumor-bearing animals described above, with the bead-based LEGENDplex™ immunoassay (BioLegend).

### Statistical analysis

Data were plotted and analyzed with Prism software (GraphPad) using two-sided tests and considering *p* < 0.05 statistically significant. ANC time course data were analyzed by two-way ANOVA with Treatment and Time as factors. G-CSF concentration, single time point ANC data and flow cytometry data were analyzed by one-way ANOVA. If the ANOVA reached *p* < 0.05, Tukey’s or Sidak’s multiple comparison test was utilized to compare groups.

## Results

### Plinabulin reduces neutropenia induced by diverse chemotherapies, with a profile different from that of a G-CSF therapy

**Fig. 1:**
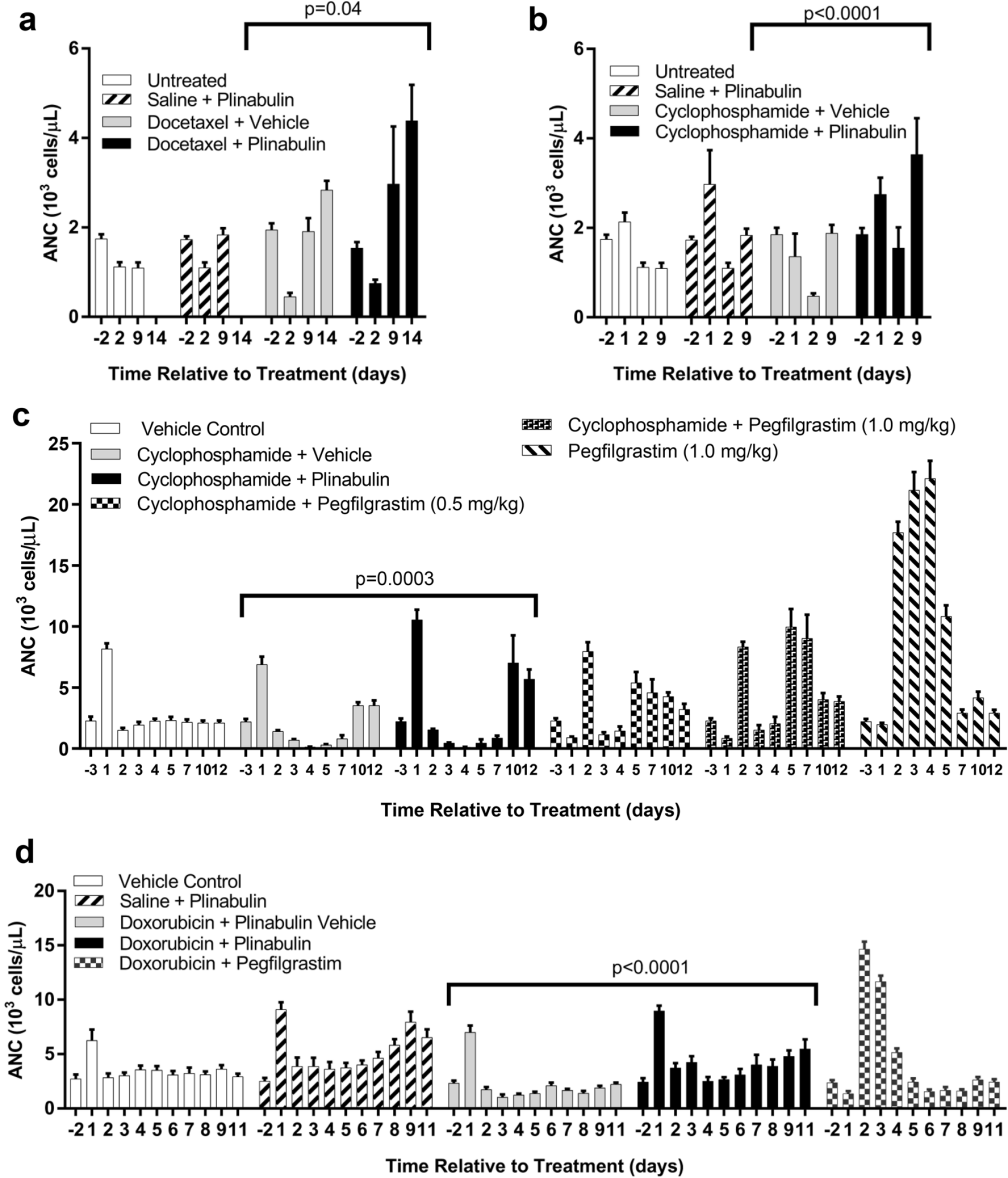
The effects of plinabulin on chemotherapy-induced neutropenia. **a** Blood absolute neutrophil count (ANC) before (− 2) and 2–14 days after intraperitoneal (IP) treatment with docetaxel (15.0 mg/kg) or 0.9% saline, followed 1 h later by plinabulin (7.5 mg/kg) or plinabulin vehicle (*n* = 5 rats/group). **b** ANC before (− 2) and 1–9 days following IP treatment with cyclophosphamide (12.5 mg/kg) or 0.9% saline, followed 1 h later by plinabulin (7.5 mg/kg) or plinabulin vehicle (*n* = 5). **c** ANC before (− 3) and 1–12 days following intravenous (IV) treatment with cyclophosphamide (50 mg/kg) or sterile water for injection, followed 1 h later by either IP plinabulin (7.5 mg/kg) or plinabulin vehicle, or 1 day later by subcutaneous (SC) dosing of pegfilgrastim (0.5 or 1.0 mg/kg). Control animals receiving only plinabulin vehicle were also included (*n* = 8). **d** ANC before (− 2) and 1–11 days following IV treatment with doxorubicin (3 mg/kg) or 0.9% saline followed either 1 h later by IP plinabulin (7.5 mg/kg) or 30 min later by plinabulin vehicle, or 1 day later by SC dosing of pegfilgrastim (0.125 mg/kg). Control animals receiving IV 0.9% saline followed 30 min later by IP plinabulin (7.5 mg/kg) or plinabulin vehicle were also included (*n* = 8). Data are presented as the mean ± SEM. Statistical *p* values indicated are for the effect of treatment by two-way ANOVA

To study whether the ability of plinabulin to reduce neutropenia induced by docetaxel in patients extends to neutropenia induced by other chemotherapies, plinabulin was administered to normal healthy rats 1 h following administration of docetaxel (tubulin-targeted anti-mitotic), cyclophosphamide (DNA cross-linking) or doxorubicin (DNA intercalation). With IP docetaxel, a transient lowering of ANC was detected 2 days after dosing, relative to untreated rats (Fig. 1a). ANC was consistently higher 2–14 days after receiving plinabulin 1 h after administration of docetaxel, compared to docetaxel monotherapy. Notably, the same pattern was observed when plinabulin was combined with IP cyclophosphamide, with reduced neutropenia 2 days after dosing and a greater rise in ANC from 2 to 9 days after dosing (Fig. 1b; *p* < 0.0001). A more detailed evaluation of the time course for ANC, utilizing a higher dose of cyclophosphamide and IV dosing, found a more dramatic rise in ANC from 7 to 12 days when plinabulin was added to cyclophosphamide (Fig. 1c). The profile of change in ANC was very different between plinabulin and pegfilgrastim when this G-CSF agent was dosed subcutaneously (SC) 24 h after chemotherapy as in patients, at two different dose levels. Notably, IP dosing of plinabulin vehicle or plinabulin caused a spike in ANC 1 day after dosing likely due to injury caused in the peritoneal cavity, emphasizing the importance of including a vehicle control group when evaluating blood neutrophil levels with IP dosing. The difference in ANC profile between plinabulin and pegfilgrastim was confirmed when these agents were combined with another chemotherapy, doxorubicin (Fig. 1d). Plinabulin prevented IV doxorubicin-induced neutropenia from 2 to 11 days after dosing (*p* < 0.0001) and again demonstrated a late rise in ANC from 5 to 11 days after treatment. On the other hand, SC pegfilgrastim at a lower dose level than previously utilized caused a dramatic increase in ANC 2–3 days after chemotherapy dosing, with little effect from 5 to 11 days. ANC effect profiles for pegfilgrastim alone (Fig. 1c) and plinabulin alone (Fig. 1d) were clearly different.

### Plinabulin does not increase bone marrow or plasma G-CSF

**Fig. 2:**
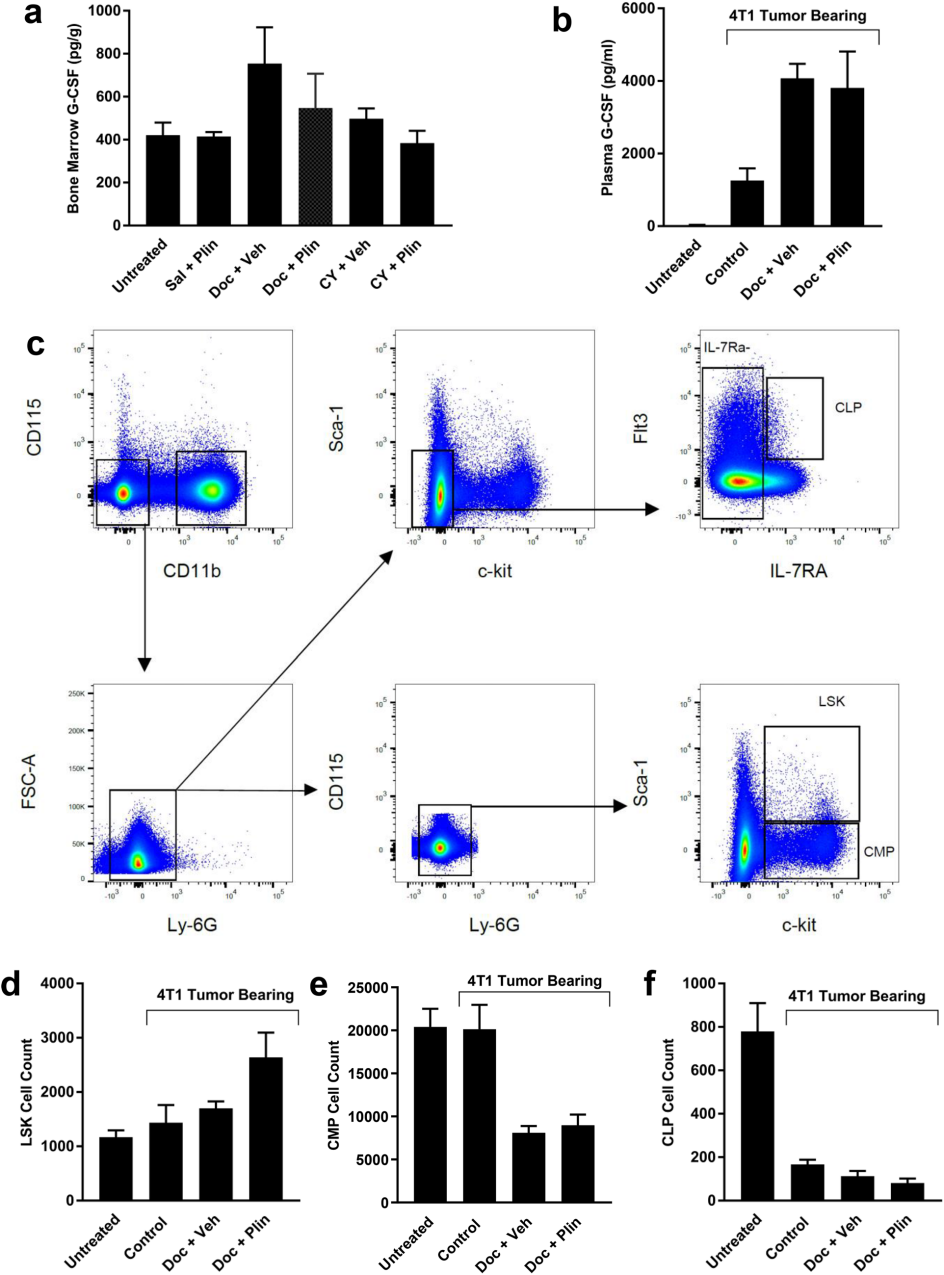
Primitive hematopoietic cells but not neutrophils or G-CSF levels were affected by the addition of plinabulin to docetaxel. **a** Bone marrow G-CSF protein concentration (pg per gram total protein) was evaluated 2 days after intraperitoneal (IP) treatment of rats with docetaxel (Doc; 15.0 mg/kg), cyclophosphamide (CY; 12.5 mg/kg) or 0.9% saline (Sal), followed 1 h later by IP plinabulin (Plin; 7.5 mg/kg) or plinabulin vehicle (Veh). **b** Plasma G-CSF was evaluated in 4T1 tumor-bearing mice 2 days after a 15 min intravenous infusion of docetaxel (22 mg/kg) or docetaxel vehicle (7.5% ethanol/7.5% Tween-80), followed 15 min later by IP injection of plinabulin (7.5 mg/kg) or plinabulin vehicle (Veh) twice, 3 h apart. Control animals received both vehicles. Untreated non-tumor-bearing mice were also included (*n* = 5). **c** Illustration of the flow cytometry gating strategy for identifying CD45^+^ Lineage^−^ LSK (CD11b^−^CD115^−^Ly6G^−^Sca-1^+^c-kit^+^), common myeloid progenitor (CMP; CD11b^−^CD115^−^Ly6G^−^Sca-1^−^c-kit^+^CD16/32^−^) and common lymphoid progenitor (CLP; Sca-1^lo^c-kit^lo^Flt3^+^IL-7Rα^+^) cells in bone marrow from a control tumor-bearing mouse. **d** Total CD45-positive LSK cells isolated from both femurs of 4T1 tumor-bearing or non-tumor-bearing mice by flow cytometry, 2 days after treatment. **e** Total CMP cell counts in both femurs. **f** Total CLP cell number in both femurs. Data are presented as the mean ± SEM for *n* = 5 per group

Based on the differing patterns of ANC change with plinabulin versus various dose levels of pegfilgrastim when given in combination with chemotherapy, it is unlikely that plinabulin acts through a mechanism similar to that of pegfilgrastim. To strengthen this conclusion, rat femur bone marrow G-CSF levels were measured 2 days after chemotherapy dosing, when ANC was reduced (Figs. 1a, b). Docetaxel, but not cyclophosphamide, tended to increase G-CSF at this time point (Fig. 2a; *p* = 0.12 for ANOVA). Plinabulin alone or in combination with chemotherapy had no effect on bone marrow G-CSF levels. To incorporate the potential effect of a tumor on G-CSF levels [17], the effect of plinabulin on plasma G-CSF levels was tested in 4T1 murine breast cancer tumor-bearing mice (Fig. 2b). G-CSF was clearly elevated 2 days after dosing in vehicle-treated tumor-bearing mice compared to untreated non-tumor-bearing mice, and docetaxel more than doubled this value (*p* = 0.0045). Plinabulin in contrast had no effect on plasma G-CSF when added to docetaxel treatment (*p* = 0.98).

### LSK hematopoietic stem progenitor cells are increased when plinabulin is added to docetaxel

Chemotherapy is known to target bone marrow cells to induce neutropenia, and it is from the bone marrow that recovery or protection may originate through hematopoiesis. In an unpublished work, plinabulin reduced neutropenia induced by docetaxel in non-tumor-bearing C57BL/6 mice [Ghosh et al., AACR 2018, Abstract 4805], with a mechanism thought to involve increased production of neutrophils in the bone marrow, possibly by relieving a docetaxel-induced accumulation of murine HSPC (Lin^−^Sca1^+^c-Kit^+^ or LSK cells) observed 5 days after treatment. 5-FU [18, 19], cyclophosphamide + Ara-C [2], or LPS [20] administration in mice induced increased bone marrow LSK cell numbers 1–2 days after treatment, indicating that cell stress-induced effects on LSK number may occur more rapidly than 5 days. LSK cells in murine bone marrow were therefore evaluated 2 days after docetaxel and docetaxel plus plinabulin treatment to determine if plinabulin could also boost LSK cell number (Fig. 2c).

Neither the presence of tumors nor treatment with docetaxel significantly affected the total number of LSK cells (Fig. 2d; *p* > 0.59), despite changes in G-CSF (Fig. 2b). However, when plinabulin was added to docetaxel, LSK cell number was significantly increased compared to untreated non-tumor-bearing mice (*p* = 0.01) and vehicle-treated tumor-bearing mice (*p* = 0.048). The trend for an increase compared to docetaxel-treated mice did not reach statistical significance (*p* = 0.15). In contrast to LSK cells, more differentiated common myeloid progenitor cells (CMP; Fig. 2e) and common lymphoid progenitor cells (CLP; Fig. 2f) were reduced in tumor-bearing mice (*p* < 0.0001) and their levels tended to be further reduced 2 days after docetaxel monotherapy. Adding plinabulin to docetaxel had no significant effect on total bone marrow CLP or CMP cell numbers (*p* > 0.96) at 2 days after treatment, nor other cells relevant to the myeloid lineage (Online Resource 2).

## Discussion

Neutropenia and associated infection are life-threatening side effects of cancer chemotherapy. CIN is also a major cause for chemotherapy dose reductions and delays that may compromise cancer treatment outcomes. Use of recombinant G-CSF has transformed management of neutropenia in the clinic, yet CIN and its treatment remain significant concerns in the delivery of cancer chemotherapy. Plinabulin has been shown to significantly limit neutropenia in NSCLC patients when combined with docetaxel, but the ability to reduce neutropenia induced by non-tubulin-targeted chemotherapy has not been reported. Although species differences exist in the details of the hematopoietic hierarchy from stem cells to mature lineage cells [16], animal models allow for discovery and hypothesis testing in a manner often not possible in humans. Here, we show in nonclinical models that plinabulin not only boosts blood neutrophil counts in combination with a microtubule stabilizing agent, but does so with DNA cross-linking and DNA intercalating chemotherapies as well. Moreover, the effects of plinabulin on ANC were unlike those of pegfilgrastim and not associated with an increase in G-CSF, supporting the potential of plinabulin to address the need for non-G-CSF-based anti-CIN therapies.

Although effective against CIN, warnings for G-CSF therapies include allergic reactions, splenic rupture, acute respiratory distress, alveolar hemorrhage and hemoptysis, with bone pain in 10–30% of patients [5]. These effects have not been reported with plinabulin, whose adverse effects are primarily gastrointestinal [9]. In animal CIN models as well, plinabulin exhibited a different profile of ANC effects compared to that of pegfilgrastim. Moreover, plinabulin did not affect endogenous G-CSF levels in bone marrow or plasma in the models tested. Data therefore indicate that plinabulin may serve as an alternative therapy to G-CSF for prophylactic CIN therapy and should also be considered for testing in combination with G-CSF therapies, especially in settings where adequate ANC control by G-CSF therapy is lacking [e.g., 8].

The ability of plinabulin to increase ANC may be related to positive effects on cells of the hematopoietic system. Bone marrow generation of mature blood cell lineages proceeds through successive differentiation from HSPC to more highly proliferative progenitor cells and leukocyte precursor cells [16, 18, 19]. Chemotherapies treat cancer by targeting proliferating cancer cells but also cause neutropenia and other hematopoiesis-related adverse immunological effects by inadvertently targeting dividing bone marrow cells [2, 21]. Although total HSPC numbers in murine bone marrow, consisting of cycling and dormant HSPCs, are reportedly reduced in animals by some chemotherapy regimens, beginning 2 days after treatment of C57BL/6 mice with 5-FU or cyclophosphamide + Ara-c, for example [2, 18, 19], dormant LSK cells switch to self-renewal 1–2 days following chemotherapy in the same studies. In C57BL/6 [22] and Balb/c mice [20], despite the reported inter-strain differences in the magnitude of Sca1 expression on HSPCs [23], lipopolysaccharide (LPS) boosts LSK cell number by 24 h after treatment in both mouse strains. The boost to LSK cell number by chemotherapy and LPS may be related to the ability of stem/progenitor cells to respond to stressors such as chemotherapy, infection, or inflammation, by entering the cell cycle and increasing their proliferation rate [19, 22], and/or by shifting the balance of HSPC towards LSK cells [20], in preparation for increased hematopoiesis. Here we have shown that while the presence of tumors or treatment with docetaxel did not significantly affect LSK number in the bone marrow of mice, adding plinabulin to docetaxel in tumor-bearing mice resulted in a significant increase in LSK cell number within 2 days. Interestingly, the finding that both plinabulin and LPS [20, 22] increased bone marrow LSK number in Balb/c mice is reminiscent of their common ability to mature dendritic cells, possibly indicating overlapping molecular signaling pathways [13].

The consistent increase in ANC found in plinabulin-treated rats, beginning approximately 7 days after therapy, may be related to the 1–2 weeks necessary to form mature neutrophils from HSPCs [3, 4]. Moreover, following differentiation of the LSK cells, whose numbers were increased 2 days after docetaxel + plinabulin treatment in mice, it is conceivable that by 5 days after treatment with docetaxel plus plinabulin, the LSK cell number in bone marrow may be reduced compared to docetaxel alone, as reported in non-tumor-bearing C57BL/6 mice [Ghosh et al., AACR 2018, Abstract 4805]. It is important to study the effects of plinabulin further in this regard, since a therapy that increases HSPC number has the potential to alleviate chemotherapy-induced deficiencies in multiple mature cell populations in the hematopoietic system. Indeed, unpublished data indicate plinabulin alleviates docetaxel-induced thrombocytopenia as well as neutropenia in NSCLC patients [Blayney et al., IASCLC 2018, Abstract P1.01-06]. Additional support for an effect on stem/progenitor cells in human subjects derives from the finding that plinabulin caused a dose-dependent increase in the number of circulating white blood cells positive for CD34 [Blayney et al., ASH 2018, *Blood* 132 (Supplement 1):2068], a marker for hematopoietic stem and progenitor cells in human that is reportedly low or negative on murine hematopoietic stem LSK cells [24].

In summary, plinabulin has beneficial effects on chemotherapy-induced neutropenia induced by chemotherapies of different classes, with a mechanism distinct from G-CSF-based therapies. Results reported here support the continued development of plinabulin as an alternative and/or combinatorial approach to G-CSF therapy for the treatment of CIN.

## Electronic supplementary material

Below is the link to the electronic supplementary material.

Online Resource 1 Dose-dependent effects of plinabulin on doxorubicin-induced neutropenia. Blood absolute neutrophil count (ANC) 2 days after intraperitoneal treatment with plinabulin (7.5 mg/kg), or intravenous treatment with doxorubicin (3 mg/kg), followed 1 h later by intraperitoneal plinabulin (1.75, 3.5 or 7.5 mg/kg) or plinabulin vehicle (n = 6 rats/group). Data are presented as the mean ± SEM. Statistical *p* value indicated is for the effect of treatment by one-way ANOVA (PDF 119 kb)

Online Resource 2 Effects of treatment on bone marrow cells involved in myeloid lineage hematopoiesis. **a** Gating strategy example for flow cytometry analyses of bone marrow collected from both femurs of untreated mice or 4T1 tumor-bearing mice, 2 days after a 15 min intravenous infusion of docetaxel (22 mg/kg; Doc) or docetaxel vehicle (7.5% ethanol/7.5% Tween-80), followed 15 min later by IP injection of plinabulin (7.5 mg/kg) or plinabulin vehicle (Veh) twice, 3 h apart. Control tumor-bearing animals received both vehicles (gating strategy example shown). Total number of, **b** CD45 + Lineage-multipotent progenitors (MPP; CD48-Sca-1 + c-kithiFlt3 + CD150−), **c** common myeloid progenitors (CMP; CD11b-CD115-Ly6G-Sca-1-c-kit + CD16/32−), **d** granulocyte/macrophage progenitors (GMP; IL-7Rα^−^Sca-1^−^c-kit^+^ CD34^+^ CD16/32^+^), **e** neutrophils (CD115-CD11b + Ly6Ghi) and **f** monocytes (c-kit-CD115^+^) collected from both femurs. Data are presented as the mean ± SEM for n = 5 mice per group (PDF 304 kb)
