# Supplementary material for: Plinabulin ameliorates neutropenia induced by multiple chemotherapies through a mechanism distinct from G-CSF therapies
Source: Cancer Chemother Pharmacol. 2019 Dec 6;85(2):461–8. doi: 10.1007/s00280-019-03998-w (PMC7015961; doi:10.1007/s00280-019-03998-w)

Article Title: Plinabulin Ameliorates Neutropenia Induced by Multiple Chemotherapies Through a Mechanism Distinct from G-CSF Therapies

Cancer Chemotherapy and Pharmacology

Authors: James R. Tonra, G. Kenneth Lloyd, Ramon Mohanlal, Lan Huang
Corresponding Author: James R. Tonra, Ph.D., BeyondSpring Pharmaceuticals, jtonra@beyondspringpharma.com

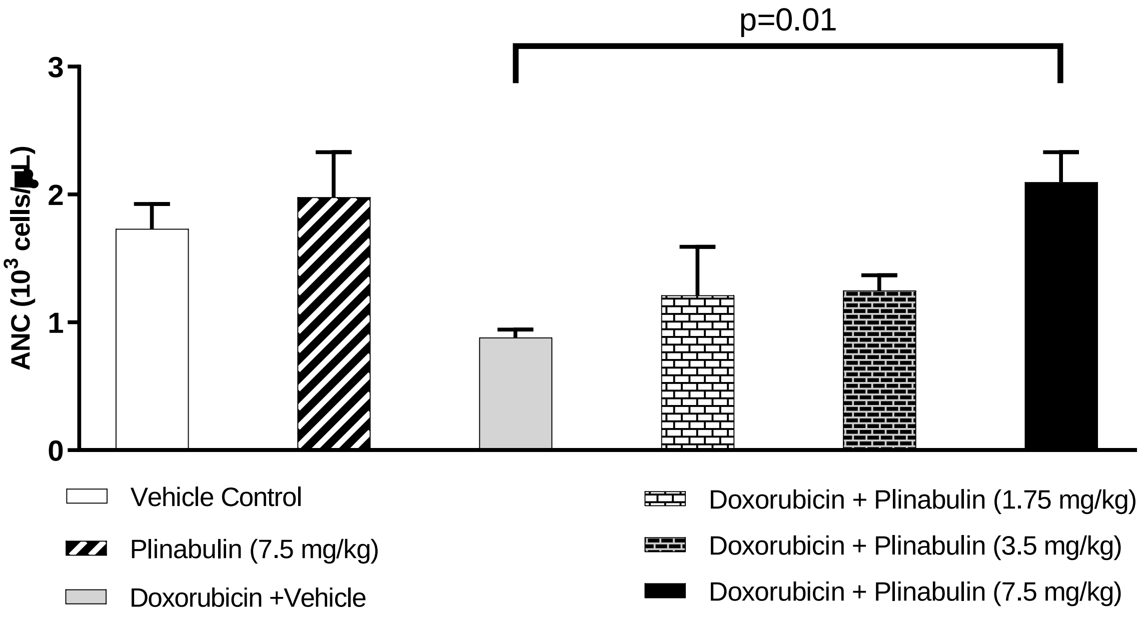

Supplement: Supplementary file 1 — Online Resource 1 Dose-dependent effects of plinabulin on doxorubicin-induced neutropenia. Blood absolute neutrophil count (ANC) 2 days after intraperitoneal treatment with plinabulin (7.5 mg/kg), or intravenous treatment with doxorubicin (3 mg/kg), followed 1 h later by intraperitoneal plinabulin (1.75, 3.5 or 7.5 mg/kg) or plinabulin vehicle (n = 6 rats/group). Data are presented as the mean ± SEM. Statistical p value indicated is for the effect of treatment by one-way ANOVA (PDF 119 kb) [file 280_2019_3998_MOESM1_ESM.pdf]
