# Supplementary material for: Plinabulin ameliorates neutropenia induced by multiple chemotherapies through a mechanism distinct from G-CSF therapies
Source: Cancer Chemother Pharmacol. 2019 Dec 6;85(2):461–8. doi: 10.1007/s00280-019-03998-w (PMC7015961; doi:10.1007/s00280-019-03998-w)
Supplement: Supplementary file 2 — Online Resource 2 Effects of treatment on bone marrow cells involved in myeloid lineage hematopoiesis. a Gating strategy example for flow cytometry analyses of bone marrow collected from both femurs of untreated mice or 4T1 tumor-bearing mice, 2 days after a 15 min intravenous infusion of docetaxel (22 mg/kg; Doc) or docetaxel vehicle (7.5% ethanol/7.5% Tween-80), followed 15 min later by IP injection of plinabulin (7.5 mg/kg) or plinabulin vehicle (Veh) twice, 3 h apart. Control tumor-bearing animals received both vehicles (gating strategy example shown). Total number of, b CD45 + Lineage-multipotent progenitors (MPP; CD48-Sca-1 + c-kithiFlt3 + CD150−), c common myeloid progenitors (CMP; CD11b-CD115-Ly6G-Sca-1-c-kit + CD16/32−), d granulocyte/macrophage progenitors (GMP; IL-7Rα−Sca-1−c-kit+ CD34+ CD16/32+), e neutrophils (CD115-CD11b + Ly6Ghi) and f monocytes (c-kit-CD115+) collected from both femurs. Data are presented as the mean ± SEM for n = 5 mice per group (PDF 304 kb) [file 280_2019_3998_MOESM2_ESM.pdf]

Article Title: Plinabulin Ameliorates Neutropenia Induced by Multiple Chemotherapies Through a Mechanism Distinct from G-CSF Therapies

Cancer Chemotherapy and Pharmacology

Authors: James R. Tonra, G. Kenneth Lloyd, Ramon Mohanlal, Lan Huang

Corresponding Author: James R. Tonra, Ph.D., BeyondSpring Pharmaceuticals, jtonra@beyondspringpharma.com

a

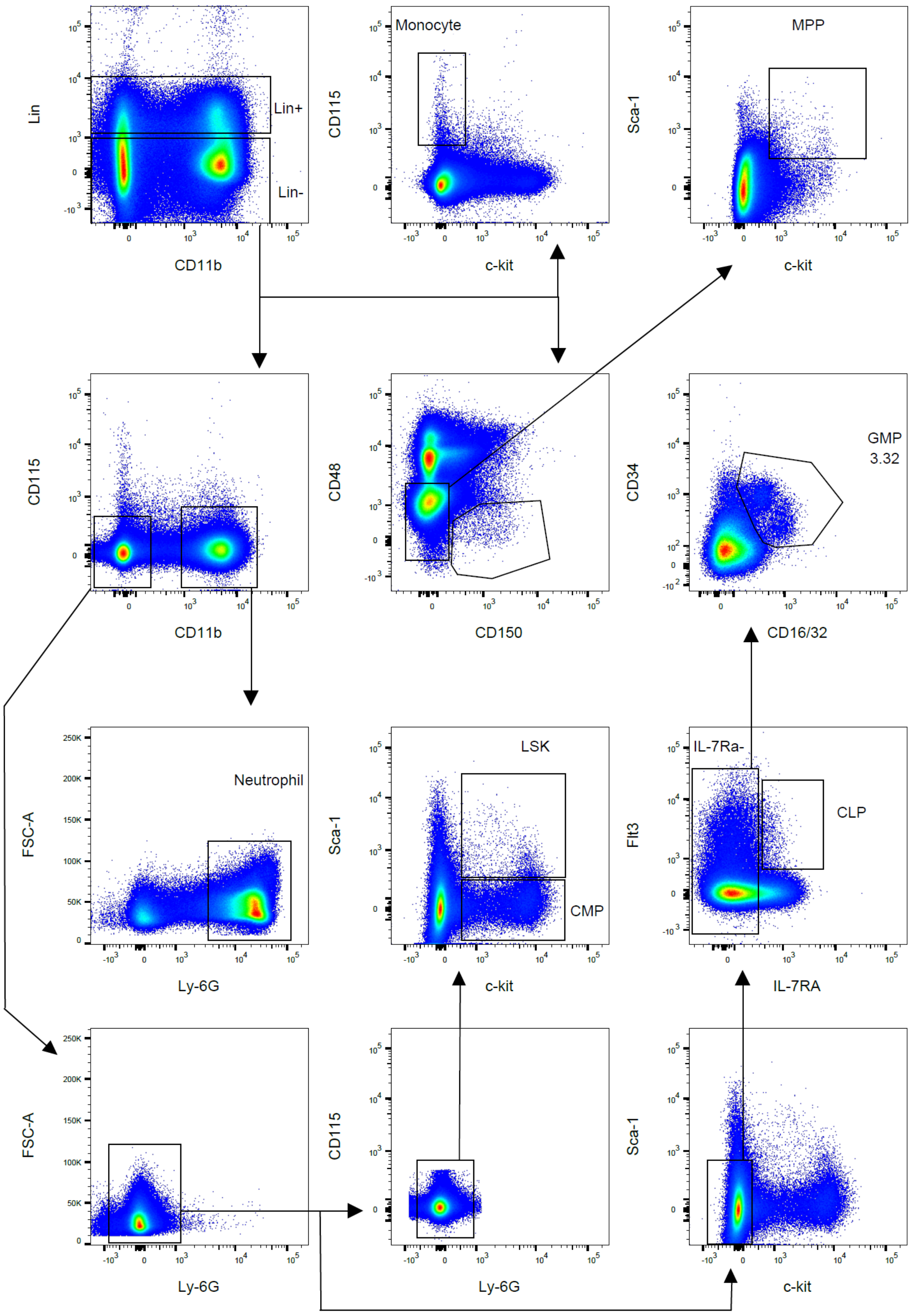

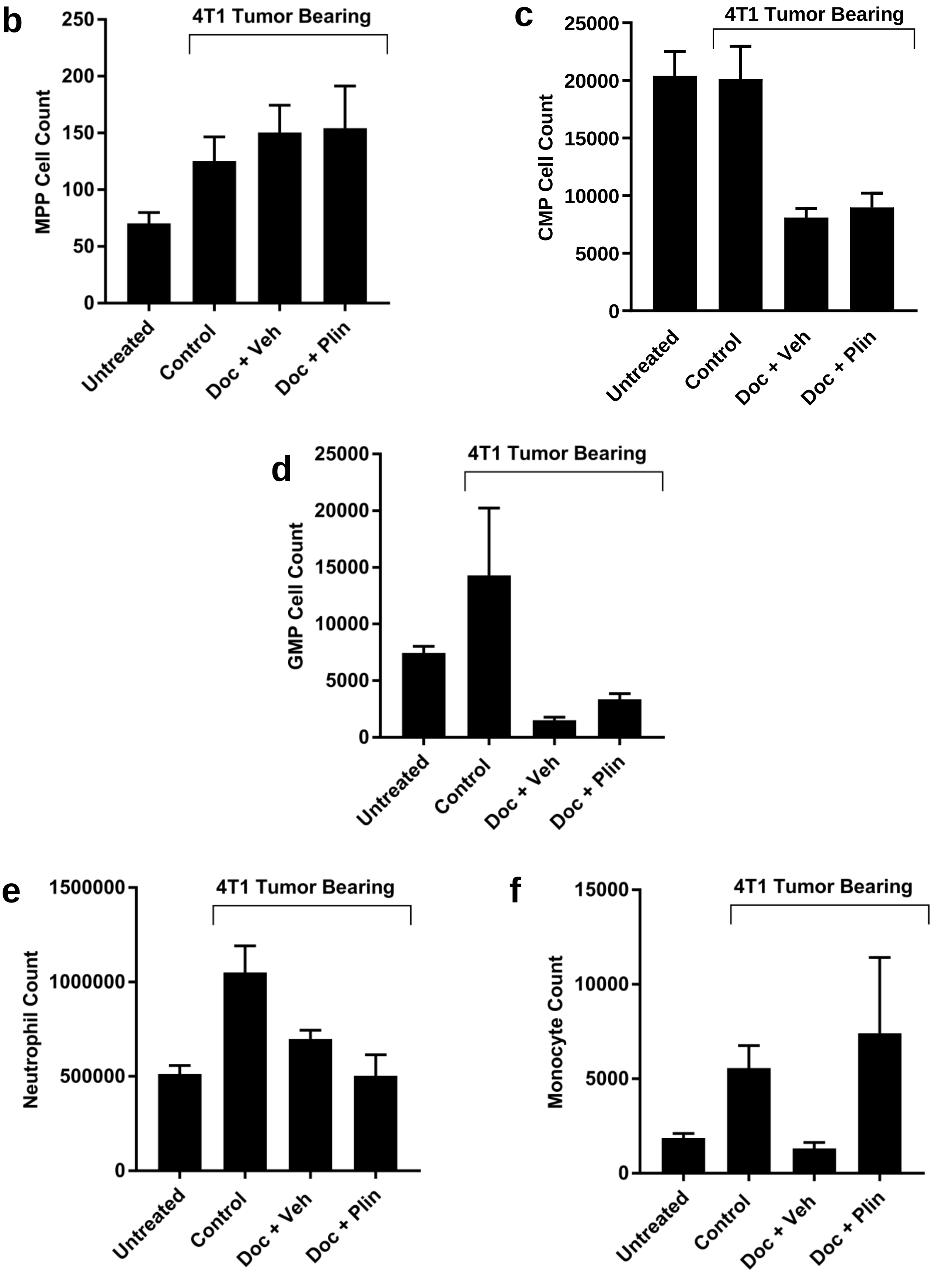
b
4T1 Tumor Bearing
MPP Cell Count
250
200
150
100
50
0
Untreated
Control
Doc + Veh
Doc + Plin
c
4T1 Tumor Bearing
CMP Cell Count
25000
20000
15000
10000
5000
0
Untreated
Control
Doc + Veh
Doc + Plin
d
4T1 Tumor Bearing
GMP Cell Count
25000
20000
15000
10000
5000
0
Untreated
Control
Doc + Veh
Doc + Plin
e
4T1 Tumor Bearing
Neutrophil Count
1500000
1000000
500000
0
Untreated
Control
Doc + Veh
Doc + Plin
f
4T1 Tumor Bearing
Monocyte Count
15000
10000
5000
0
Untreated
Control
Doc + Veh
Doc + Plin
